# Fas Apoptosis Inhibitory Molecule Blocks and Dissolves Pathological Amyloid-β Species

**DOI:** 10.3389/fnmol.2021.750578

**Published:** 2021-12-14

**Authors:** Hiroaki Kaku, Alexander V. Ludlow, Michael F. Gutknecht, Thomas L. Rothstein

**Affiliations:** ^1^Center for Immunobiology, Kalamazoo, MI, United States; ^2^Department of Investigative Medicine, Western Michigan University Homer Stryker M.D. School of Medicine, Kalamazoo, MI, United States

**Keywords:** fas apoptotic inhibitory molecule (FAIM), amyloid-β, Alzheimer’s disease, protein aggregate, protein disaggregation by chaperones

## Abstract

A number of neurodegenerative diseases are associated with the accumulation of misfolded proteins, including Alzheimer’s disease (AD). In AD, misfolded proteins such as tau and amyloid-β (Aβ) form pathological insoluble deposits. It is hypothesized that molecules capable of dissolving such protein aggregates might reverse disease progression and improve the lives of afflicted AD patients. Here we report new functions of the highly conserved mammalian protein, Fas Apoptosis Inhibitory Molecule (FAIM). We found that FAIM-deficient Neuro 2A cells accumulate Aβ oligomers/fibrils. We further found that recombinant human FAIM prevents the generation of pathologic Aβ oligomers and fibrils in a cell-free system, suggesting that FAIM functions without any additional cellular components. More importantly, recombinant human FAIM disaggregates and solubilizes established Aβ fibrils. Our results identify a previously unknown, completely novel candidate for understanding and treating irremediable, irreversible, and unrelenting neurodegenerative diseases.

## Introduction

Dementia is a progressive, incurable, and uniformly fatal neurodegenerative disorder (Ballard et al., 2011). Most forms of dementia are associated with both cognitive and non-cognitive impairments that can severely affect patients’ quality of life (Gale et al., 2018). The prevalence of dementia increases with age (Prince et al., 2013; Baumgart et al., 2015). Alzheimer’s disease (AD) is the primary cause of progressive dementia, followed by Lewy body dementia (LBD) and frontotemporal dementia (FTD) (Sancesario and Bernardini, 2018). A common, pathological hallmark of these dementias is the accumulation of misfolded proteins into insoluble proteinaceous deposits in and around affected tissues (Lovestone and McLoughlin, 2002). In most cases, the major constituent of an insoluble aggregate is a disease-specific protein, such as amyloid-β (Aβ) in AD, α-synuclein (α-syn) in LBD, or TAR DNA-binding protein 43 (TDP-43) in FTD. Additionally there is ample evidence emerging of protein interaction between the dementias, such as hyper-phosphorylated tau neurofibrillary tangles (encoded by *MAPT* gene) in both AD and FTD (McAlary et al., 2019). Although it was initially accepted that these insoluble protein fibrils play a key role in disease etiology and pathophysiology, a growing number of studies suggest that soluble oligomers or protofibrils can be more neurotoxic than mature insoluble fibrils (Usenovic et al., 2015; Verma et al., 2015; Fusco et al., 2017; Cline et al., 2018; Siddiqi et al., 2019). It is thought that altered protein homeostasis involving disease-associated misfolded proteins is caused by reduced chaperone activity or reduced clearance of aggregated proteins by autophagy (Komatsu et al., 2006; Loeffler, 2019). Concomitant with aging, genetic (mutation and polymorphism), and environmental (cellular stress and exposure to metals) factors may cause protein misfolding and the accumulation of protein oligomers/fibrils (Kikis et al., 2010; Gidalevitz et al., 2011). Furthermore, both oligomers and fibrils provide templates to seed the further oligomerization/fibrilization of monomers, and can spread from neuron to neuron *via* a prion-like process (Danzer et al., 2009; Lee et al., 2010; Luk V. et al., 2012; Luk V. M. et al., 2012; Mougenot et al., 2012; Stohr et al., 2012; Masuda-Suzukake et al., 2013; Brettschneider et al., 2015; Condello and Stoehr, 2018). Therefore, recent research has focused on understanding cellular processing of oligomeric and fibrillar protein aggregates involved in neurodegenerative diseases. While the exact role of misfolded proteins in these pathologies is still under debate, the deleterious formation of soluble oligomers and protofibrils, and insoluble mature fibrils is an ideal target for future disease-modifying therapies for Alzheimer’s disease and related dementias (ADRD).

Fas apoptosis inhibitory molecule is a 20-kDa cytosolic protein composed of 179 amino acids; it was originally discovered in 1999 and was thought to be a FAS-apoptosis inhibitor based on its identification in Fas-resistant B cells and on overexpression studies in a B cell line (Schneider et al., 1999). A subsequent study identified an alternatively spliced form, termed FAIM-Long (L), which has 22 additional amino acids at the N-terminus (Zhong et al., 2001). Thus, the originally identified FAIM was renamed FAIM-Short (S). Although two other gene products were confusingly termed FAIM2 (also termed lifeguard) and FAIM3 (also termed TOSO) by other groups (Planells-Ferrer et al., 2016), neither FAIM2 nor FAIM3 are related to FAIM-S and FAIM-L in terms of function or protein homology. FAIM-L is expressed almost exclusively in the brain whereas FAIM-S is ubiquitously expressed (Zhong et al., 2001). In the brain, FAIM-S is expressed in astrocytes, microglia and neurons. FAIM-L is only expressed in neurons but at higher levels than FAIM-S (Segura et al., 2007; Coccia et al., 2017) (hereafter FAIM indicates both FAIM-S and FAIM-L). The physiological role of FAIM has been enigmatic for many years because of the absence of any homologous protein sequences, or any recognizable consensus effector/binding motifs; with limited structural information on the full peptide it has been difficult to predict FAIM’s authentic functions. Recent work showed that FAIM opposes aggregation of a mutant protein associated with some hereditary forms of ALS, suggesting a role in proteostasis (Kaku et al., 2020). However, it is unclear whether this activity extends to other aggregation-prone proteins and/or other disease states; in particular, whether and to what extent FAIM can antagonize pathogenic Aβ species remains unexplored. In this study, we seek to understand the activity of FAIM for Aβ using both a cellular and an *in vitro* cell-free system.

## Materials and Methods

### Reagents and Antibodies

β-lactoglobulin B (βLG) from bovine milk (18.2 kDa) (#L8005) and bovine serum albumin (BSA) (66.5 kDa) (#A7906) were purchased from MilliporeSigma, and used as negative control proteins. Rabbit anti-amyloid-β (#8243), mouse-anti-amyloid precursor protein (APP) (#2452), goat anti-rabbit IgG-HRP-linked (#7074) and horse anti-mouse IgG-HRP-linked (#7076) antibodies were obtained from Cell Signaling Technology. Affinity purified anti-FAIM antibody was obtained from rabbits immunized with CYIKAVSSRKRKEGIIHTLI peptide (located near the C-terminal region of FAIM) as previously described (Kaku and Rothstein, 2009a,b).

### Cell Culture and Transfection

The Neuro 2A (N2a) cell line was obtained from the American Type Culture Collection (ATCC, #CCL-131). N2a cells were cultured in DMEM/F12 medium (Cytiva) containing 10% FCS, 15 mM HEPES, 2.5 mM L-glutamine and 0.1 mg/ml penicillin and streptomycin. Transfection was performed using Lipofectamine 2000 according to the manufacturer’s instructions (Invitrogen). In brief, N2a cells were seeded at 1 × 10^5^ cells/well in 24-well plates, and transfected 24 h later with the indicated DNA plasmid (0.5 μg/well) using Lipofectamine 2000 transfection reagent (1 μl/well). pcDNA FRT TO- APP695 and pcDNA FRT TO- APPSwed/Ind were gifts from Aleksandra Radenovic (Addgene plasmids #114193 and #114194, respectively). pcDNA3.3 plasmid (Themo Fisher Science, #K830001) was obtained from Invitrogen, and was used as a negative control (Empty).

### Generation of Fas Apoptosis Inhibitory Molecule Knockout Neuro 2A Cells With CRISPR/Cas9

Guide RNA (gRNA) sequences for mouse *faim* gene were designed using a CRISPR target design tool^1^ in order to target the exon after the start codon as previously described (Kaku and Rothstein, 2020). Briefly, designed DNA oligo nucleotides were ligated into pSpCas9(BB)-2A-GFP (PX458) vector (Addgene plasmid #48138, a gift from Feng Zhang) at the Bpi1 (Bbs1) restriction enzyme sites using the “Golden Gate” cloning strategy. The presence of insert was verified by sequencing. Empty vector was used as a negative control. After fourth passage, transfection was performed using lipofectamine 2000 as above and a week after the transfection, eGFP^+^ cells were single-sorted with a Melody instrument (Becton Dickinson), and seeded into 96 well plates. FAIM knockout clones were screened by limiting dilution and western blotting. The cells were expanded for three passages and afterward frozen in 90% FBS and 10% DMSO (Sigma-Aldrich, # 472301) at passage seven in liquid nitrogen for future use. After thawing cells, the cells with total 10 passages were used for the further experiments.

### Cell Viability Analysis With Flow Cytometry

Neuro 2A cells were detached by Trypsin-EDTA. Adherent and floating cells were harvested and pooled, after which cells were resuspended in 2 μg/ml 7-aminoactinomycin D (7-AAD) (Anaspec, #AS-83201). Cell viability was assessed using an Attune flow cytometer (Thermo Scientific). Data were analyzed using v10 software (TreeStar).

### His-Tag Recombinant Protein Production

His-tag protein expression vectors were constructed using pTrcHis TA vector (Themo Fisher Science, #K441001) according to the manufacturer’s instructions as previously described (Kaku et al., 2020). In brief, PCR amplified target genes were TA-cloned into the pTrcHis TA vector (Themo Fisher Science) and inserted DNA was verified by sequencing (Genewiz). Oligonucleotides used in this work for PCR-cloning into pTrcHis TA vector were as follows: human *FAIM-S* forward, “ATGACAGATCTCGTAGCTGT TTGG”; human *FAIM-S* reverse, “TTAACTTGCAATCTCTG GGATTTC”; human *FAIM-L* forward, “ATGGCATCTGG AGATGACAGTC”; human *FAIM-L* reverse, “TTAACTTGCAA TCTCTGGGATTTC”; human *HSP27* forward, “ATGACC GAGCGCCGCGTCCCCTT”; human *HSP27* reverse, ‘’TTA CTTGGCGGCAGTCTCATCGGAT.”

The sequence primer used for the pTrcHis-TA vector is as follows: TATGGCTAGCATGACTGGT.” Proteins were expressed in TOP10 competent cells (Themo Fisher Science, #C404010) with IPTG (Invitrogen, #15529019) at 1 mM for 2.5 h at 37°C, and were purified using a Nuvia IMAC Nickel-charged column (Bio-Rad) on an NGC Quest chromatography system (Bio-Rad). Proteins underwent dialysis against PBS using Slide-A-Lyzer Dialysis Cassettes, 10K MWCO (Themo Fisher Science, #66380). Protein purity was verified using TGX Stain-Free gels (Bio-Rad) on ChemiDoc Touch Imaging System (Bio-Rad) and each protein was determined to be >90% pure (Supplementary Figure 1). Random mutagenesis was performed using GeneMorpho II Random Mutagenesis Kit (Agilent, #200550) according to the manufacturer’s recommendation. Briefly, pTrcHis TA vector containing human FAIM-S gene was used as a template, and was amplified for 30 cycles using mutazyme II. Oligonucleotides used in PCR-mutagenesis were as follows: human *FAIM-S* forward, “GATGACGATAAGGATCCAACCCTTATG”; human *FAIM-S* reverse, ‘’AGCTTCGAATTGAATTCGCCCTTTTA.” After PCR-amplified DNA was re-inserted into the pTrcHis TA vector, the insert was verified by sequencing, and proteins were expressed as above.

### Thioflavin T Fluorescence Assay

Fibril/aggregate formation of 5 μM Aβ (1–42) (Aβ42) (Anaspec, #AS-72216), assembled in a 384-well clear bottom plate (Corning), was assessed by 50 μM Thioflavin T (ThT) (MilliporeSigma, #T3516) fluorescence using a Synergy Neo2 Multi-Mode Microplate Reader (Bio-Tek). Reader temperature was set at 37°C with continuous shaking between reads. ThT fluorescence intensity was measured using an excitation wavelength of 440 nm and an emission of 482 nm. PMT gain was set at 80. Fluorescence measurements were made from the bottom of the plate, with the top being sealed with an adhesive plate sealer to prevent evaporation.

### Generation of Pre-formed Aβ Fibrils

Preformed Aβ42 fibrils were assembled by incubating 5 μM Aβ42 monomers in amyloid-β assay buffer (Anaspec) for 1 h with agitation at 559 rpm at 37°C. Fibrils were recovered by centrifugation at 14,000 × *g* for 10 min, washed and resuspended in PBS. Fibril generation was confirmed by 50 μM ThT fluorescence. Fibrils were diluted to the requisite concentration for subsequent disaggregation reactions.

### Disaggregation Assay by ThT, Filter Trap Assay and Sedimentation Analysis

Aβ42 (0.5 μM) pre-formed fibrils were incubated with indicated recombinant proteins at 37°C for 2.5 h. Then, fibril status was determined either by ThT fluorescence, by filter trap assay (FTA), or by analysis of supernatants and pellets after sedimentation and western blotting (sedimentation analysis). For FTA, samples underwent vacuum filtration through a pre-wet 0.2 μm pore size nitrocellulose membrane (GE Healthcare) using a 96 well format Dot-Blot apparatus (Bio-Rad). The membrane was washed twice with PBS and western blotting using anti-Aβ antibody was carried out to detect Aβ fibrils.

### SDS-PAGE and Western Blotting

Total protein samples were subjected to sodium dodecyl-sulfate polyacrylamide gel electrophoresis (SDS-PAGE) on an Any kD Mini-PROTEAN TGX Precast Protein Gel (Bio-Rad) followed by immunoblotting after wet transfer for 1 h to PVDF membrane (Bio-Rad) and blocking with 1% non-fat dry milk (Bio-Rad) in TBS-T. In some experiments (Figures 3D, 4A), protein samples were centrifuged at 14,000 × *g* for 10 min. Pellets were washed by PBS, and suspended in 1x Laemmli buffer. Both supernatant and pellet fractions were subjected to SDS-PAGE. Densitometry quantification of the bands was analyzed by ImageLab software (version 6.1.0) (Bio-Rad). For Supplementary Figure 1, 2, stain-free technology was used to detect total proteins using the ChemiDoc Touch Imaging System (Gilda and Gomes, 2013).

### Statistical Analysis

All quantitative data are expressed as mean ± SEM. A two-tailed, unpaired Student’s *t*-test was used for statistical determinations with GraphPad Prism 8 and 9 software. Values of *p* < 0.05 are considered statistically significant (*p < 0.05, ^**^p < 0.01 or ^***^p < 0.001.

## Results

### Aβ Fibrils/Aggregates Accumulate in Fas Apoptosis Inhibitory Molecule-Deficient Neuro 2A Cells Following Amyloid Precursor Protein-Overexpression

**FIGURE 1:**
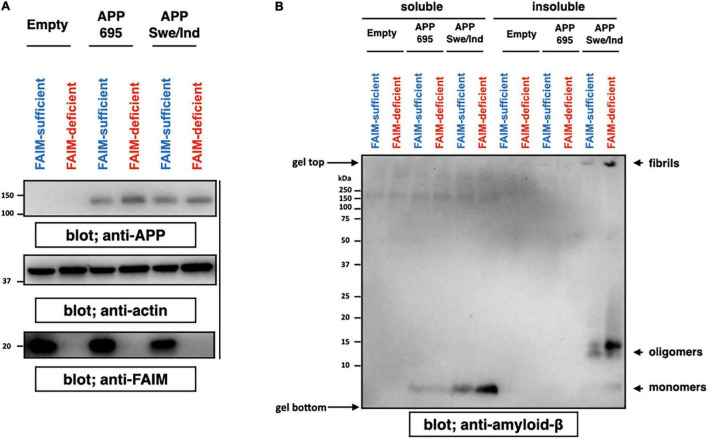
FAIM KO cells accumulate insoluble Aβ fibrils/aggregates. WT and FAIM KO Neuro 2A cells were transiently transfected with expression vectors for APP695 and mutant APPswe/ind. Four days later after the transfection, cells and culture supernatants were harvested. **(A)** Cell lysates were prepared using RIPA buffer, and were analyzed by western blotting for APP, FAIM, and actin as a loading control. **(B)** Culture supernatants were fractionated into soluble and insoluble pellet fractions by centrifugation at 14,000 × *g* for 10 min. Pellets were washed twice with PBS. Proteins from the supernatants for each fraction were analyzed by western blotting for Aβ. Representative data from at least three independent experiments are shown.

We previously showed ubiquitinated protein aggregates induced by cellular stress and mutant SOD1 aggregates accumulate in FAIM-deficient cells (Kaku et al., 2020; Kaku and Rothstein, 2020). To assess the role FAIM plays in prevention of Aβ aggregation, we employed an APP-overexpression system using Neuro 2A cells, a mouse neuroblastoma. It is reported that mutant APP such as Swedish mutant (K670N/M671L) and Indiana mutant (V717F) produce extracellular Aβ fibrils/aggregates due to abnormal processing by endogenous secretase pathways in Neuro 2A cells (Ghosh and Osswald, 2014). We transiently overexpressed APPswe/ind and control APP695 into WT and FAIM-deficient Neuro 2A cells (Figure 1A), and found that cell cultures with mutant APP contained aggregated Aβ 4 days after the transfection (Figure 1B) as previously reported (Park et al., 2015; Cuddy et al., 2017). Regardless of transfection efficiency of APP (Figure 1A), the levels of Aβ fibrils/aggregates in the culture supernatant were dramatically higher in FAIM-deficient Neuro 2A cells than in WT Neuro 2A cells (Figure 1B). The loading control for Figure 1B is shown in Supplementary Figure 2. Although we unexpectedly observed monomer and oligomer Aβ bands in the pellet-insoluble fraction in addition to mature fibrils, mature fibrils might be broken down to monomers/oligomers during the procedure (i.e., exposure to SDS) (Figure 1B). To exclude the possibility that the differences observed in Figure 1B could be due to differences of cell viability after transfection of APP, cell viability was assayed by 7-AAD staining. We did not observe a significant difference in cell death among groups (Supplementary Figure 3). These results demonstrate the essential role of FAIM in altering the fate of mutant APP-derived Aβ.

### Recombinant Human Fas Apoptosis Inhibitory Molecule Proteins Prevent the Formation of Aβ42 Soluble Protofibrils and Oligomers in a Cell-Free System

**FIGURE 2:**
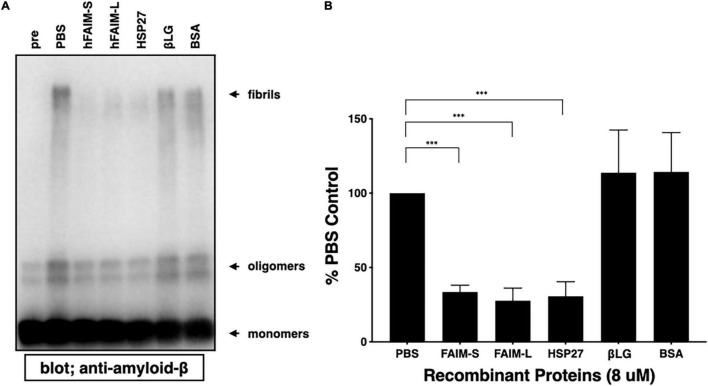
Recombinant FAIM suppresses protein fibrillization/aggregation in a cell-free system. **(A)** Spontaneous oligomerization of Aβ (15 μM) *in vitro* was induced at room temperature for 18 h in the presence of 1 μM recombinant FAIM-S, FAIM-L, HSP27, βLG or BSA. Total samples were subjected to SDS-PAGE and western blotted for Aβ. Representative data from at least three independent experiments are shown. Pre: pre-incubation, PBS: buffer control. **(B)** The fibril levels in **(A)** were quantified by densitometry, and the data from three independent experiments are shown as percent of PBS control. The data are expressed as mean ± SEM. A two-tailed, unpaired Student’s *t*-test was used to calculate *p*-values. ^***^p < 0.001.

To examine whether FAIM directly inhibits the generation of Aβ42 fibrils and oligomers in an *in vitro* cell-free system, and whether the FAIM protein alone is sufficient to inhibit protein aggregation without additional cellular components, we mixed FAIM and Aβ42 monomers and incubated the mixture at room temperature. It has been reported that a mixture of soluble Aβ42 protofibrils and oligomers are formed when monomers are incubated at room temperature without agitation (Jiang et al., 2019). We used HSP27 as a positive control, because HSP27 has been shown to inhibit Aβ42 fibrillization/aggregation in cell-free systems (Lee et al., 2006; Raman et al., 2005; Fonte et al., 2008). We found that the formation of these soluble species was prevented in the presence of recombinant FAIM or HSP27 in this condition (Figures 2A,B). These data suggest that FAIM has activity in preventing the generation of Aβ42 oligomers and fibrils.

### Recombinant Human Fas Apoptosis Inhibitory Molecule Proteins Suppress Aβ42 Fibrillization/Aggregation in a Cell-Free System

**FIGURE 3:**
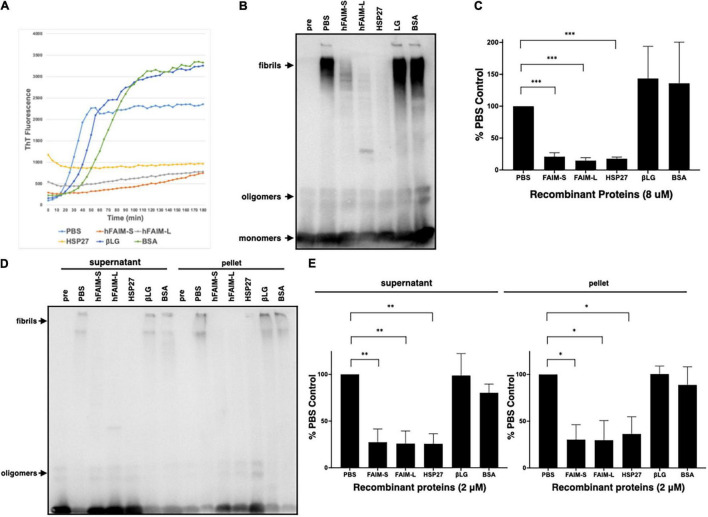
Recombinant FAIM suppresses protein fibrillization/aggregation in a cell-free system. **(A)** Spontaneous fibrillization of Aβ (5 μM) *in vitro* was monitored by ThT assay over a period of 2 h in the presence of 2 μM recombinant FAIM-S, FAIM-L, HSP27, βLG or BSA. ThT fluorescence was recorded every 5 min. **(B)** Total samples after the same reaction without ThT were subjected to SDS-PAGE and western blotted for Aβ. **(C)** The densitometry of fibrils in **(B)** from 3 independent experiments are shown as percent of PBS control. The data are expressed as mean ± SEM. **(D)** Protein samples after the same reaction without ThT were centrifuged at 14,000 × *g* for 10 min. Both supernatant and pellet fractions were subjected to SDS-PAGE and western blotted for Aβ. Representative data from at least three independent experiments are shown. **(E)** The densitometry of fibrils in **(D)** from three independent experiments are shown as percent of PBS control. The data are expressed as mean ± SEM. **(C,E)** A two-tailed, unpaired Student’s *t*-test was used to calculate *p*-values. *p < 0.05, ^**^p < 0.01 or ^***^p < 0.001.

To examine whether FAIM inhibits protein Aβ fibrillization we used Thioflavin T (ThT), a dye that only fluoresces when incorporated into amyloid fibrils (Biancalana and Koide, 2010; Malmos et al., 2017; Aliyan et al., 2019). We mixed recombinant FAIM and aggregation-prone monomeric Aβ42 peptide, and monitored aggregation status in real-time by ThT fluorescence intensity. We found that Aβ42 fibrillization was abrogated in the presence of recombinant FAIM or HSP27, but not by PBS, βLG, or BSA (Figure 3A). The normalized ThT fluorescence at 2hr is shown in Supplementary Figure 4. We performed random mutagenesis to identify a mutant form of FAIM-S lacking the activity, and found that S155N mutation abolished the activity of FAIM-S. The FAIM-S S155N mutant protein was used as a negative control in addition to BSA and βLG (Supplementary Figure 4). To confirm these results, fibrillization status was assessed by SDS-PAGE and western blotting (WB). We found aggregated Aβ42 in the high molecular weight range of negative controls (PBS, βLG, and BSA controls). We found the formation of high molecular weight aggregates was dramatically reduced in the presence of recombinant FAIM or HSP27 (Figures 3B,C). We further assessed the solubility of the samples by sedimentation in order to separate soluble protofibrils from mature insoluble fibrils. Both of them were dramatically reduced in the presence of recombinant FAIM or HSP27 (Figures 3D,E).

## Recombinant Human Fas Apoptosis Inhibitory Molecule Proteins Solubilize Preformed Aβ42 Fibrils

**FIGURE 4:**
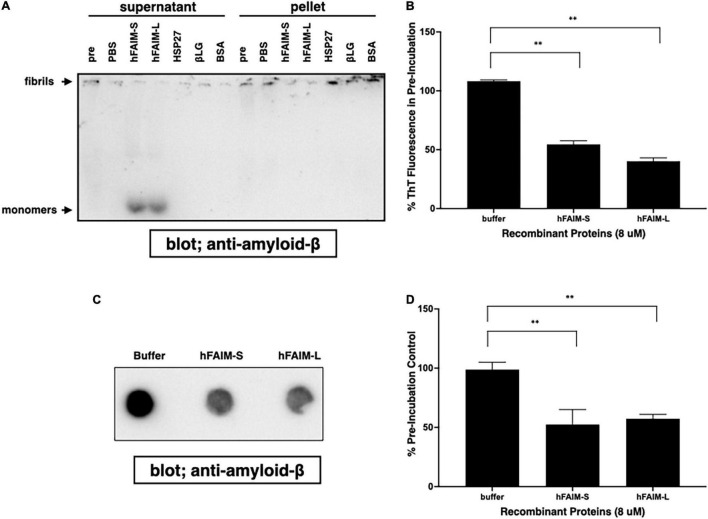
Recombinant FAIM solubilizes amyloid-β fibrils and generates monomers in a cell-free system. Pre-formed amyloid-β1-42 (Aβ42) fibrils (0.5 μM) were incubated with or without 5 μM recombinant FAIM, HSP27, βLG, BSA or PBS. for 2.5 hr followed by sedimentation analysis **(A),** Thioflavin T (ThT) staining **(B)**, and filter trap assay **(C)** to evaluate the levels of Aβ42 species. For sedimentation analysis, samples were fractionated into supernatant and pellet fractions by centrifugation at 14,000 × *g* for 10 min. Pellets were washed twice with PBS, and then native-PAGE gels were used because SDS partially solubilizes Aβ42 fibrils and produces Aβ42 monomers. Pre; pre-incubation with proteins. **(D)** The trapped fibril levels in **(C)** were quantified by densitometry, and the data from three independent experiments are shown as percent of pre-incubation control. The data are expressed as mean ± SEM. A two-tailed, unpaired Student’s *t*-test was used to calculate *p*-values. Representative data from at least three independent experiments are shown. ^**^p < 0.01.

In order to examine whether FAIM is capable of reversing pre-formed, established protein Aβ42 fibrils in addition to preventing protein aggregation, we prepared preformed amyloid-β fibrils and then added recombinant FAIM proteins. We monitored fibrillization status by differential sedimentation followed by solubilization in loading buffer and gel electrophoresis. Most of pre-formed Aβ42 fibrils alone appeared in the pellet fraction (Figure 4A). We found that the addition of FAIM led to a shift in the molecular size of the bulk of pre-formed Aβ42 fibrils that now appeared as monomers in the supernatant fractions after native-PAGE (Figure 4A). We extended these results by examining protein aggregates with complementary approaches. We prepared Aβ42 fibrils and then added recombinant FAIM proteins, as before. Fibrillization status was monitored by ThT fluorescence and by FTA. We found that ThT fluorescence (Figure 4B) and filter-trapped fibrils (Figures 4C,D) were significantly decreased after addition of FAIM as compared to negative controls. These results indicate that, in an *in vitro* cell-free system, established Aβ42 fibrils are dissolved by FAIM, at least in part.

### Recombinant Fas Apoptosis Inhibitory Molecule Suppresses Aβ Fibrillization/Aggregation in Neuro 2A Cells

**FIGURE 5:**
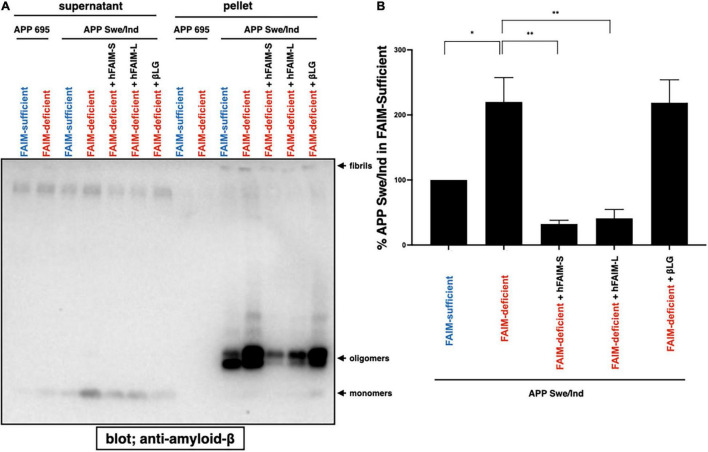
Recombinant FAIM prevents Aβ fibrils in Neuro 2A cells. **(A)** WT and FAIM KO Neuro 2A cells were transiently transfected with expression vectors for APP695 and mutant APPswe/ind. One day later, 1 μM hFAIM-S, hFAIM-L, or βLG were added into the cell culture. At day 4 after the transfection, culture supernatants were fractionated into soluble and insoluble-pellet fractions by centrifugation at 14,000 × *g* for 10 min. Pellets were washed twice with PBS. Proteins for each fraction were analyzed by western blotting for Aβ. Representative data from at least three independent experiments are shown. **(B)** The levels of detergent-insoluble aggregates in **(A)** were quantified by densitometry, and the data from three independent experiments are shown as percent of APP Swe/Ind in the FAIM-sufficent lane. The data are expressed as mean ± SEM. A two-tailed, unpaired Student’s *t*-test was used to calculate *p*-values. *p < 0.05, ^**^p < 0.01.

In order to examine whether FAIM directly inhibits Aβ fibrillization/aggregation and whether FAIM protein is sufficient to inhibit Aβ fibrillization/aggregation by itself without any additional components, we added purified recombinant human FAIM proteins into the cell culture 1 day after transfection with the expression vector for mutant APPswe/ind. Supernatant was collected and separated into soluble and insoluble-pellet fractions as performed in Figure 1. We found that the addition of recombinant FAIM proteins dramatically reduced oligomerized/fibrillized Aβ levels in the insoluble-pellet fraction (Figures 5A,B). Furthermore, the levels of Aβ fibrils/aggregates in the detergent-soluble and insoluble fractions from the cell lysates were also higher in FAIM-deficient Neuro 2A cells than in WT Neuro 2A cells, and the levels of Aβ fibrils/aggregates were reduced by the addition of FAIM proteins (Supplementary Figure 5). These data suggest that extracellular FAIM proteins can prevent the formation of both extracellular and intracellular Aβ oligomers/fibrils produced by Neuro 2A cells overexpressing disease-associated mutant APP.

## Discussion

The pathophysiology of the neurodegenerative disorder, AD, involves protein aggregates in the form of Aβ plaques and tau neurofibrillary tangles (Polanco et al., 2018). An association between AD and FAIM has been suggested (Carriba et al., 2015). FAIM-L expression was found to be impaired in the brains of AD patients, especially in the late BRAAK stages, meaning that FAIM-L expression levels are inversely correlated with the levels of pathogenic forms of tau and Aβ (Carriba et al., 2015). Given that FAIM protein prevented and solubilized Aβ fibrils, we suggest the novel hypothesis that low/no FAIM expression might be pathogenically linked to more rapid, aggressive, overwhelming Aβ fibrillization in AD patients rather than simply functioning as a marker of AD progression. Furthermore, we previously showed that oxidative stress, applied *in vivo* to intact animals, produces increased protein aggregates in FAIM-deficient mice (Kaku and Rothstein, 2020). Previous reports suggest that oxidative stress is linked to neurodegenerative diseases, presumably due to accumulation of toxic protein aggregates triggered by protein oxidation (Butterfield and Kanski, 2001; Chong et al., 2005; Chen et al., 2012). Thus, diminished FAIM expression in AD patients, combined with our previous report of increased protein aggregation in several organs after oxidative stress is delivered to FAIM-deficient mice (Kaku and Rothstein, 2020), and our current finding that FAIM prevents and disrupts Aβ oligomers and fibrils, demonstrates that FAIM is pathophysiologically relevant to current paradigms for the origin and/or progression of neurodegenerative diseases that involve disruption of proteostasis.

Whether FAIM exists in the extracellular space as a secreted, excreted, or exosome-packaged material still remains unexplored. Although Aβ oligomerization/fibrilization mainly occurs in the extracellular space, intracellular Aβ oligomerization/fibrillization within neurons from AD patients and AD model animals has also been observed (Gouras et al., 2000; Takahashi et al., 2002; Ji et al., 2016). Intraneuronal Aβ accumulation is seen before plaques develop in the brain areas first affected by AD; this appears to be among the earliest changes in AD (Gouras et al., 2000) and is associated with synaptic pathology (Takahashi et al., 2002). Further, accumulation of intraneuronal Aβ occurs before plaques in AD mouse models (Billings et al., 2005; Jawhar et al., 2012). Importantly, these AD models provide evidence that intraneuronal Aβ oligomers/fibrils may play a role in neurodegeneration, neuron loss, and extracellular amyloid plaque formation, suggesting that reduction of intraneuronal Aβ oligomers/fibrils could be a potential therapeutic goal for AD. In addition, intracellular Aβ oligomers/fibrils can spread though brain in a prion-like manner, with Aβ seed accelerating plaque formation and intercellular propagation (Olsson et al., 2018).

While there is no consensus regarding its role on AD pathology, intercellular Aβ has been proposed as an early event in AD pathogenesis and may contribute to disease progression as well (LaFerla et al., 2007; Li et al., 2007; Moon et al., 2012; Esquerda-Canals et al., 2017, 2019; Wilson et al., 2017). Whether or not extracellular FAIM exists, or if intracellular FAIM might prevent or slow intercellular Aβ seeding and spreading processes is unknown.

Prior to our discovery that FAIM can dissociate aggregated proteins, HSP104 had been shown to have this function (Parsell et al., 1994). HSP104 and its homologs exist only in the genomes of plants, bacteria, yeast and choanoflagellates, but interestingly, are absent from metazoan organisms (Erives and Fassler, 2015). In contrast, FAIM arose in the genomes of choanoflagellates and has evolved throughout holozoan species. ATP is required for disaggregation activity by HSP104 (Wendler et al., 2007; Sweeny and Shorter, 2016) whereas our work demonstrates that FAIM disaggregates proteins in the absence of ATP. In fact, there is no ATP-binding site in the FAIM protein. One can envisage that FAIM might have replaced the function of HSP104 in metazoan species to spare ATP for active movement in order to enhance the survival of multicellular organisms. Recently it has been suggested that a tripartite complex of HSP70 combined with HSP40 (J protein) and HSP110 is capable of dissociating aggregated proteins in an ATP-dependent manner (Shorter, 2011; Nillegoda et al., 2015). Thus, FAIM is unique in being a single, ATP-independent protein that prevents protein aggregation and dissociates aggregated proteins, and is the only such metazoan protein with these characteristics known at this time. From the clinical-translational standpoint, manipulation of a single FAIM protein is likely to be more feasible than manipulation of a multimember protein complex that requires ATP for function. It has been suggested that solubilization of mature Aβ fibrils is the first necessary step to treating AD with antibodies that can then hasten disposal (Murphy, 2018). Although Hsp104, which is lost from metazoa, can disaggregate proteins and could be a candidate for disaggregation therapy for neurodegenerative diseases, it might cause neuroinflammation because HSP104 is a foreign antigen, which could elicit potent, unwanted immune responses. In contrast, FAIM is highly evolutionarily conserved and is a natural protein product of humans making it an attractive possibility for therapeutic intervention. Our work provides new insights into the interrelationships between FAIM and Aβ fibrillization that one day will hopefully help in treating and understanding neurodegenerative diseases including AD and other related dementias. This has the potential to lead to species-compatible, rationally designed, preventative and therapeutic interventions.

## Data Availability Statement

The original contributions presented in the study are included in the article/Supplementary Material, further inquiries can be directed to the corresponding authors.

## Author Contributions

HK designed and performed the research, analyzed and interpreted the data, and wrote the manuscript. HK, AL, and MG performed the research. TR analyzed and interpreted the data, and edited the manuscript. All authors contributed to the article and approved the submitted version.

## Conflict of Interest

The authors declare that the research was conducted in the absence of any commercial or financial relationships that could be construed as a potential conflict of interest.

## Publisher’s Note

All claims expressed in this article are solely those of the authors and do not necessarily represent those of their affiliated organizations, or those of the publisher, the editors and the reviewers. Any product that may be evaluated in this article, or claim that may be made by its manufacturer, is not guaranteed or endorsed by the publisher.

## Footnotes

1
http://crispr.mit.edu

## Supplementary Material

Supplementary Figure 1 The purity of recombinant proteins was analyzed. 100 ng of each protein was loaded to each lane on the Stain-free gel. After SDS-PAGE, proteins were visualized by the Chemidoc Stain-free system.

Supplementary Figure 2 Total Protein levels were analyzed by the stain-free technology using the ChemiDoc Touch Imaging System. The total protein loading control is shown for Figure 1B.

Supplementary Figure 3 There is no significant difference in cell viability after the transfection of APP among groups. WT and FAIM KO Neuro 2A cells were transiently transfected with expression vectors for Empty, APP695 and mutant APPswe/ind. At day 4 after the transfection, cells were stained with 7-AAD and cell viability was analyzed by flow cytometry. Representative flow data **(A)** and a summary of pooled data from 3 independent experiments **(B)** are shown. The data are expressed as mean ± SEM.

Supplementary Figure 4 Recombinant FAIM suppresses protein fibrillization/aggregation in a cell-free system. Spontaneous fibrillization of Aβ (5 μM) *in vitro* was monitored by ThT assay over a period of 2 h in the presence of 2 μM recombinant FAIM-S, FAIM-S S155N, FAIM-L, HSP27, βLG or BSA. The normalized ThT fluorescence levels at 2 hr are expressed as mean ± SEM. A two-tailed, unpaired Student’s *t*-test was used to calculate *p*-values.

Supplementary Figure 5 Recombinant FAIM prevents intracellular Aβ fibrils in Neuro 2A cells. **(A)** WT and FAIM KO Neuro 2A cells were transiently transfected with expression vectors for APP695 and mutant APPswe/ind. One day later, 1 μM hFAIM-S, hFAIM-L, or βLG were added into the cell culture. At day 4 after the transfection, cells were harvested. Cell lysates were prepared using RIPA buffer, and were fractionated into detergent-soluble and detergent-insoluble pellet fractions by centrifugation at 14,000 × *g* for 10 min. Pellets were washed twice with PBS. Proteins from each fraction were analyzed by western blotting for Aβ, and actin as a loading control. Representative data from at least three independent experiments are shown.
