# Supplementary figures and images for: Fas Apoptosis Inhibitory Molecule Blocks and Dissolves Pathological Amyloid-β Species
Source: Front Mol Neurosci. 2021 Dec 14;14:750578. doi: 10.3389/fnmol.2021.750578 (PMC8712662; doi:10.3389/fnmol.2021.750578)

Figure S1

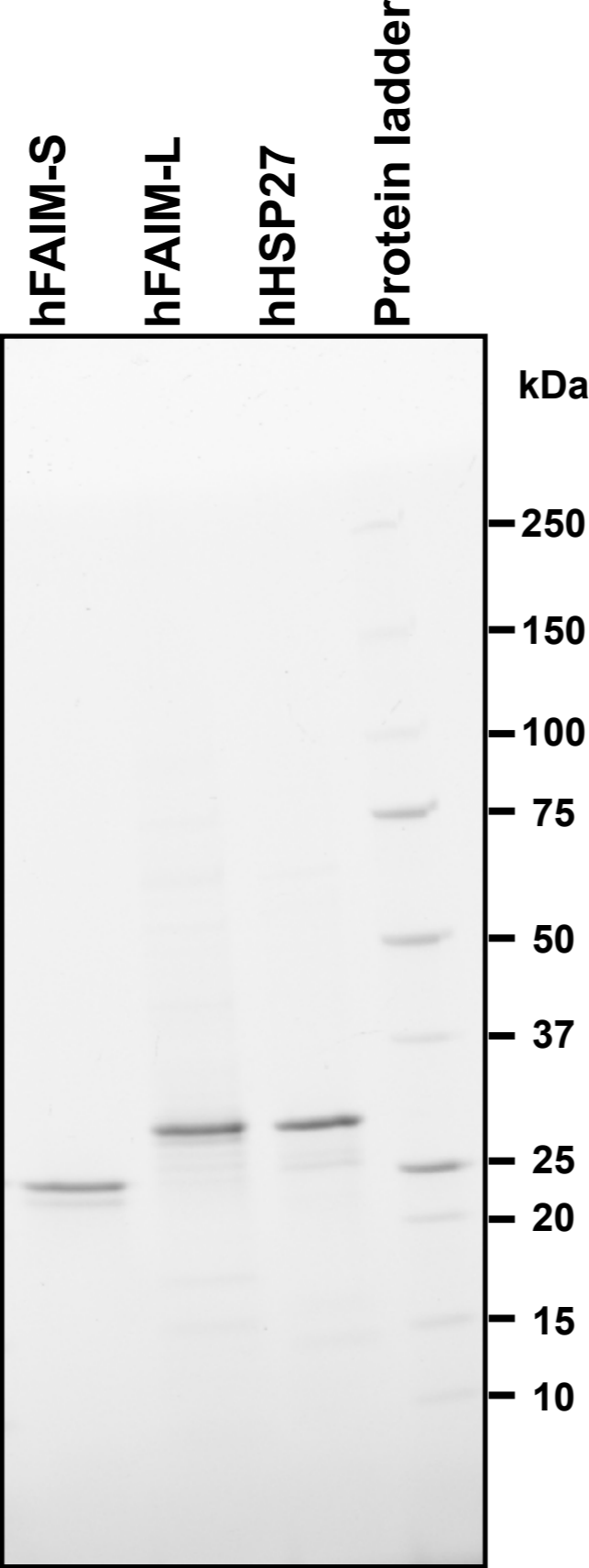

Figure S2

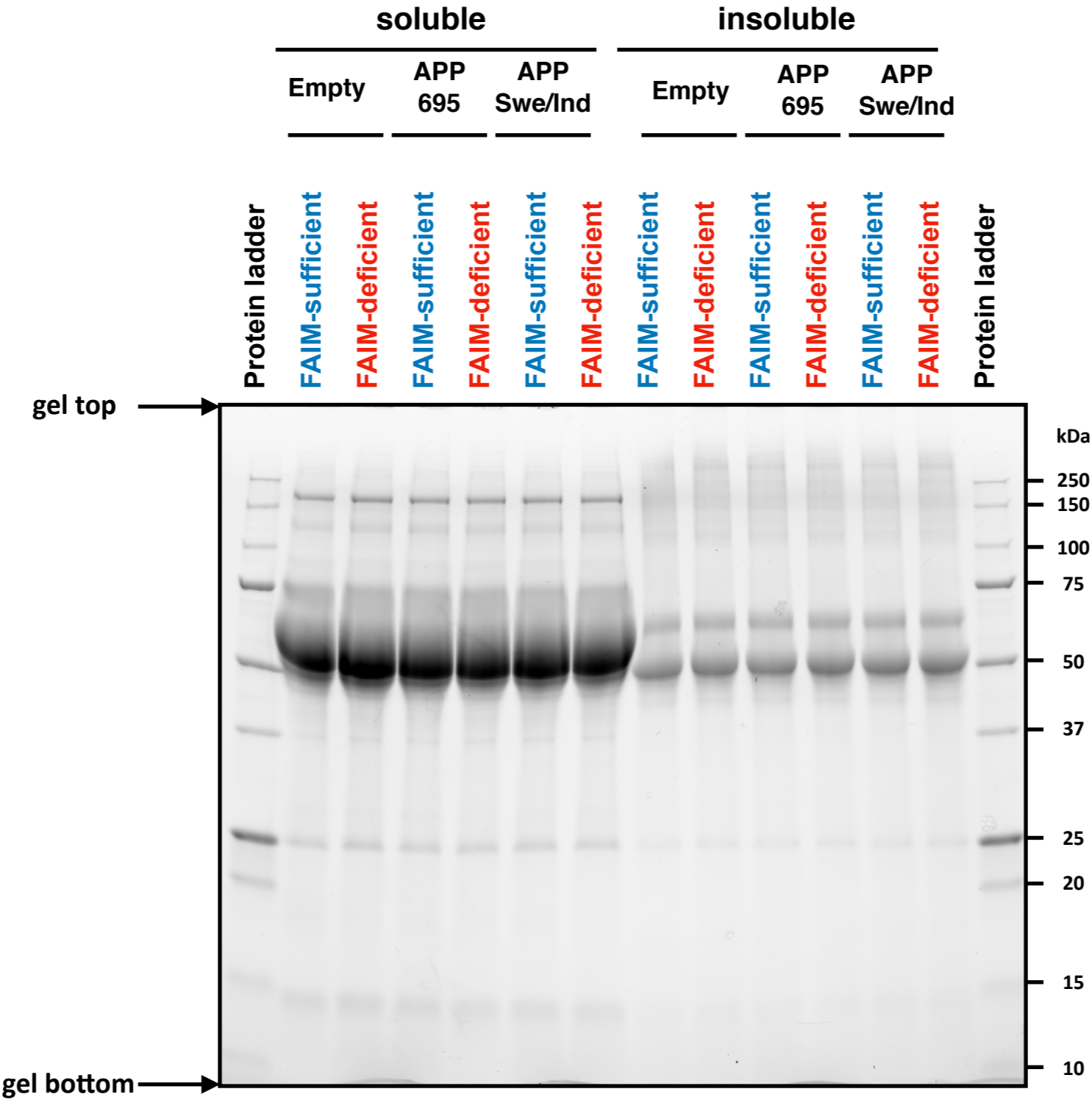

**Figure S3**

**A**

**FAIM-sufficient**

**FAIM-deficient**

**7AAD**

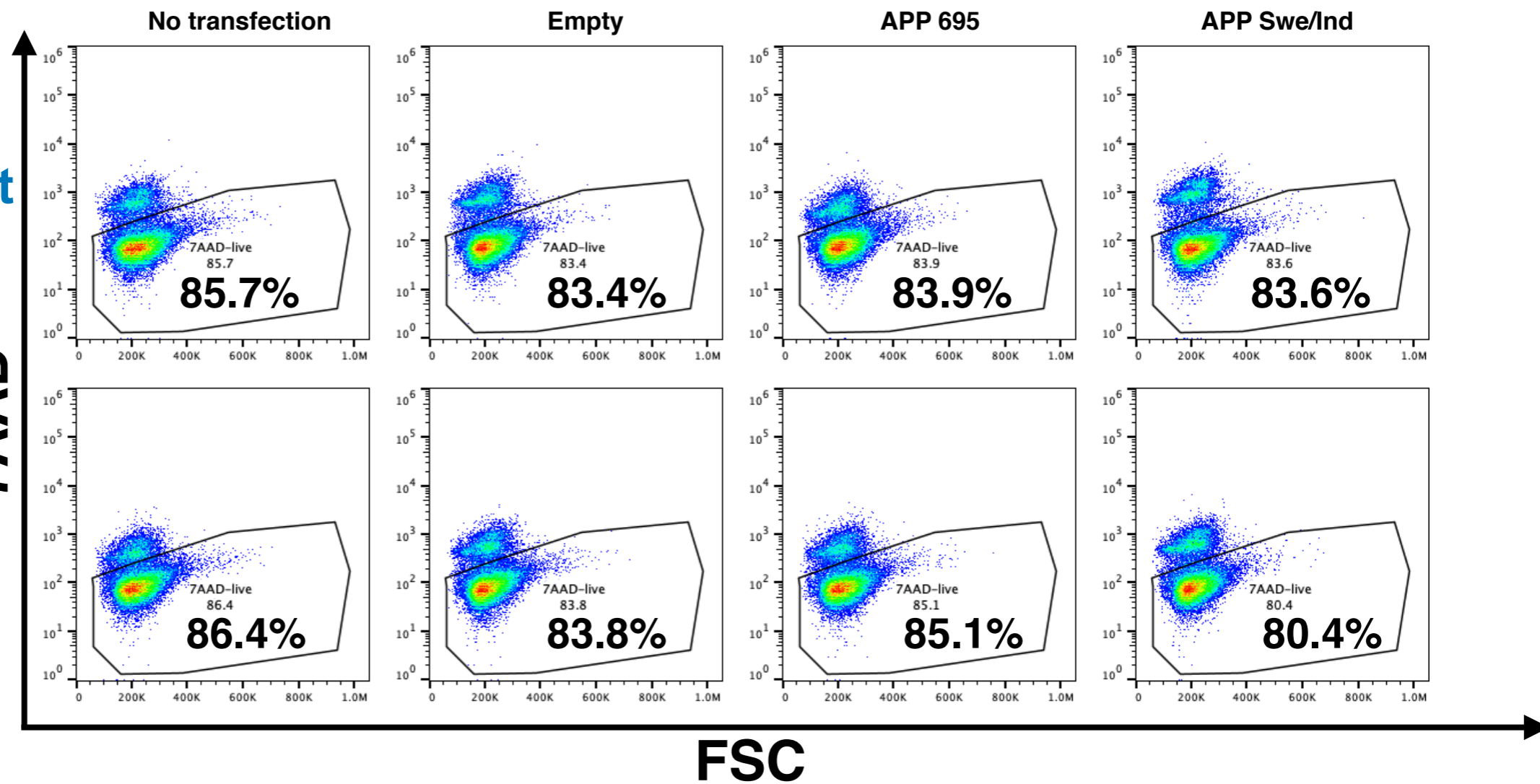

**B**

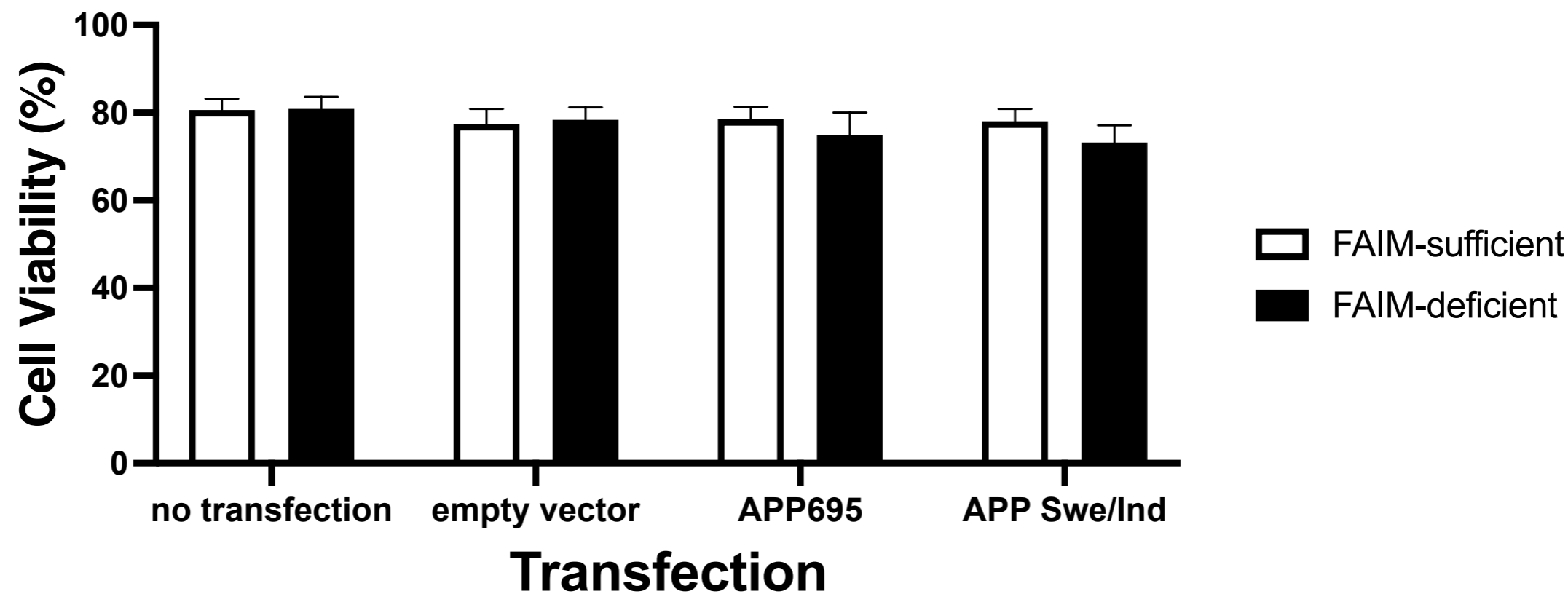

Figure S4

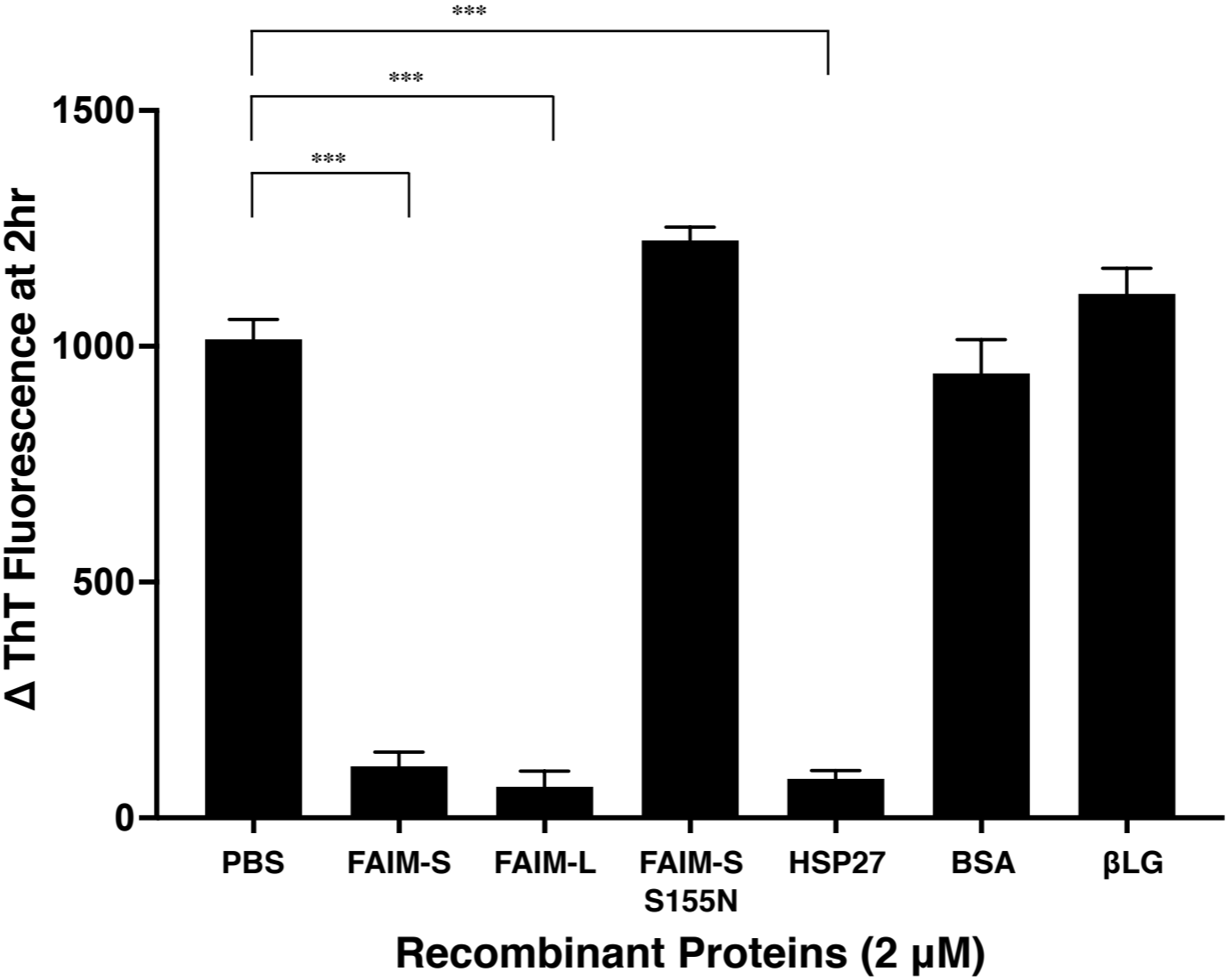

Figure S5

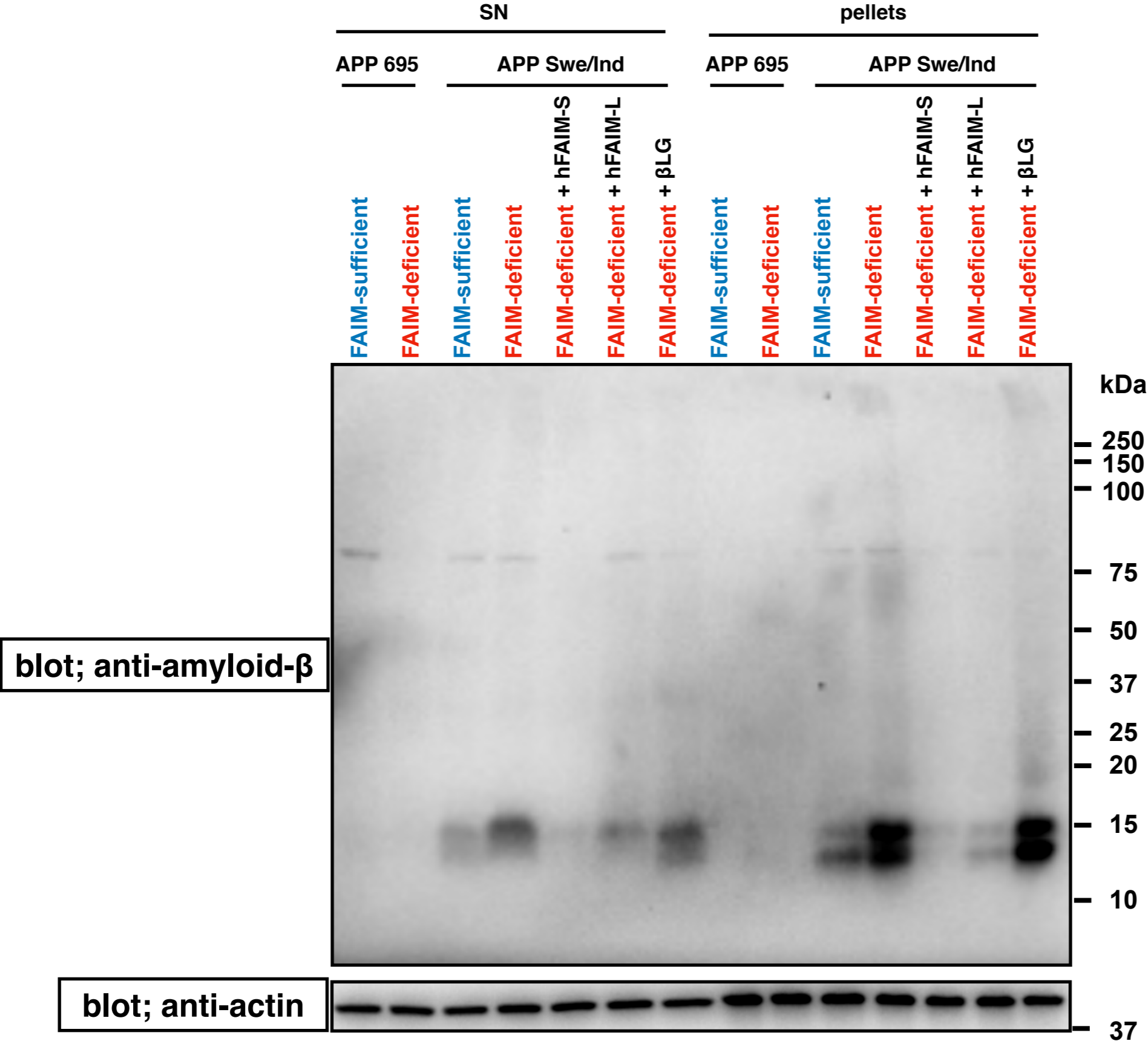

Supplement: Supplementary Figure 1 — The purity of recombinant proteins was analyzed. 100 ng of each protein was loaded to each lane on the Stain-free gel. After SDS-PAGE, proteins were visualized by the Chemidoc Stain-free system. [file Image_1.pdf]
